# Supplementary material for: Photoswitching mechanism of a fluorescent protein revealed by time-resolved crystallography and transient absorption spectroscopy
Source: Nat Commun. 2020 Feb 6;11:741. doi: 10.1038/s41467-020-14537-0 (PMC7005145; doi:10.1038/s41467-020-14537-0)
Supplement: Supplementary file 1 — Supplementary Information [file 41467_2020_14537_MOESM1_ESM.pdf]

Supplementary Information

Photoswitching mechanism of a fluorescent protein
revealed by time-resolved crystallography
and transient absorption spectroscopy

J. Woodhouse *et al*.

Supplementary table and figures

Supplementary Table 1: Data collection and refinement statistics

Dataset	laser_off	laser_on_Δ10ns
Pre-illumination (488 nm)	yes	yes
Pump-laser excitation (400 nm)	no	yes
Nominal pump-probe delay	n.a.	10 ns
Space group	$P2_12_12_1$	$P2_12_12_1$
Unit cell parameters<br>a (Å)<br>b (Å)<br>c (Å)	 51.7<br>62.9<br>71.7	 52.0<br>62.9<br>72.1
Collected frames	~609,000	~609,000
Indexed frames	9,997	9,781
Resolution (Å)	29.46 – 1.60 (1.66 – 1.60)	31.30 – 1.85 (1.92 – 1.85)
R_{split} (%)	11.64 (69.48)	10.17 (51.45)
CC*	0.996 (0.813)	0.997 (0.870)
I / σ(I)	6.8 (2.85)	7.99 (2.92)
Completeness (%)	99.87 (88.25)	99.83 (98.14)
Multiplicity	235 (5)	303 (7)
R_{iso} (with respect to *laser_off* dataset)*	n.a	0.16
Refinement statistics		
Refinement	Classical refinement	Difference-refinement
PDB entry code	6T39	6T3A#
Resolution (Å)	1.60 (1.66-1.60)	1.85 (1.90-1.85)
Number of reflections	31252 (1832)	18058 (1431)
R_{free}	20.24 (42.89)	28.67 (32.00)
R_{work}	16.53 (38.15)	23.28 (30.50)
Number of protein atoms	2338	2251
Number of water molecules	258	177
B-factor protein (Å^2)	20.2	13.4
r.m.s.d. bond lengths (Å)	0.013	0.012
r.m.s.d. angles (°)	1.37	0.614
Ramachandran favoured	98.71	98.00
Ramachandran allowed	0.86	2
Ramachadran outliers	0.43	0
Rotamer outliers	2.71	2.20
C-beta outliers	0	0
Clashscore	7.6	5.6

*R_{iso} was calculated using Phenix up to 1.85 Å resolution.

#Data deposited with PDB entry code 6T3A correspond to F_{ext}. F_{obs} of the laser_on_Δ10ns data set are available upon request.

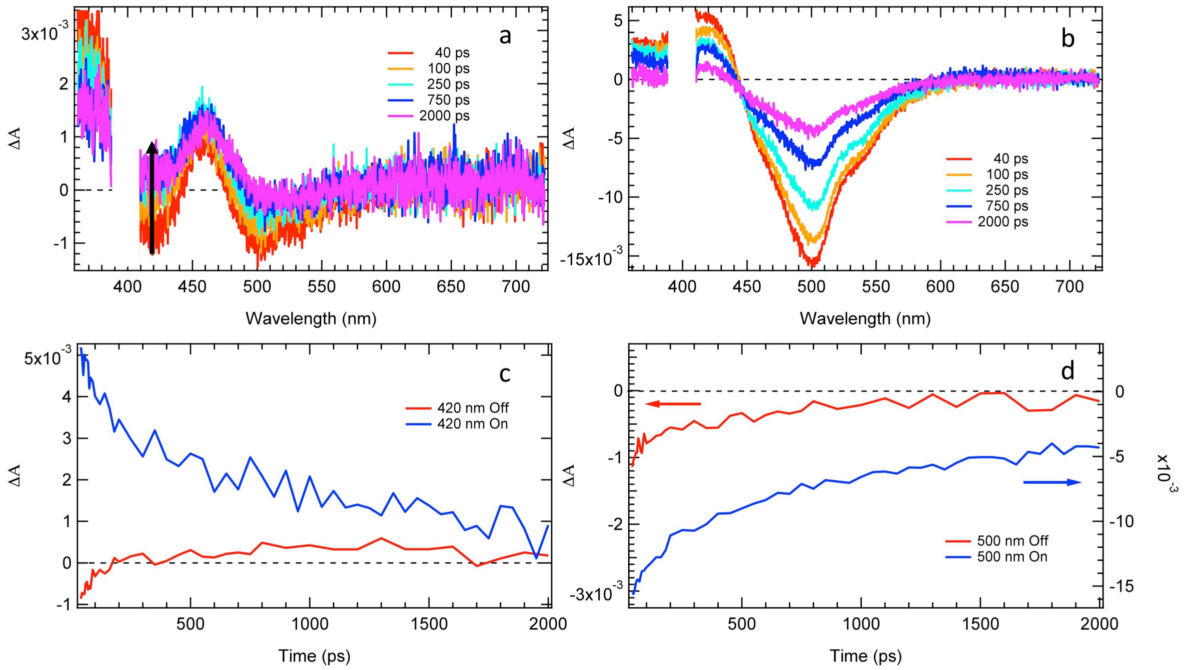

Supplementary Figure 1: Time-resolved difference absorption spectra recorded between 40 ps and 2 ns after a 400 nm femtosecond excitation of the *trans* protonated off-state (*a*) and the *cis* anionic *on* state (*b*) of rsEGFP2 in H_2O solution (50 mM HEPES pH 8, 50 mM NaCl). The spectrum without laser excitation was subtracted to calculate the difference spectra. The black arrow (*a*) indicates the disappearance of the 420 nm band within 87 ps. Kinetic traces at 420 nm (*c*) and 500 nm (*d*) extracted from panels *a* and *b* are shown.

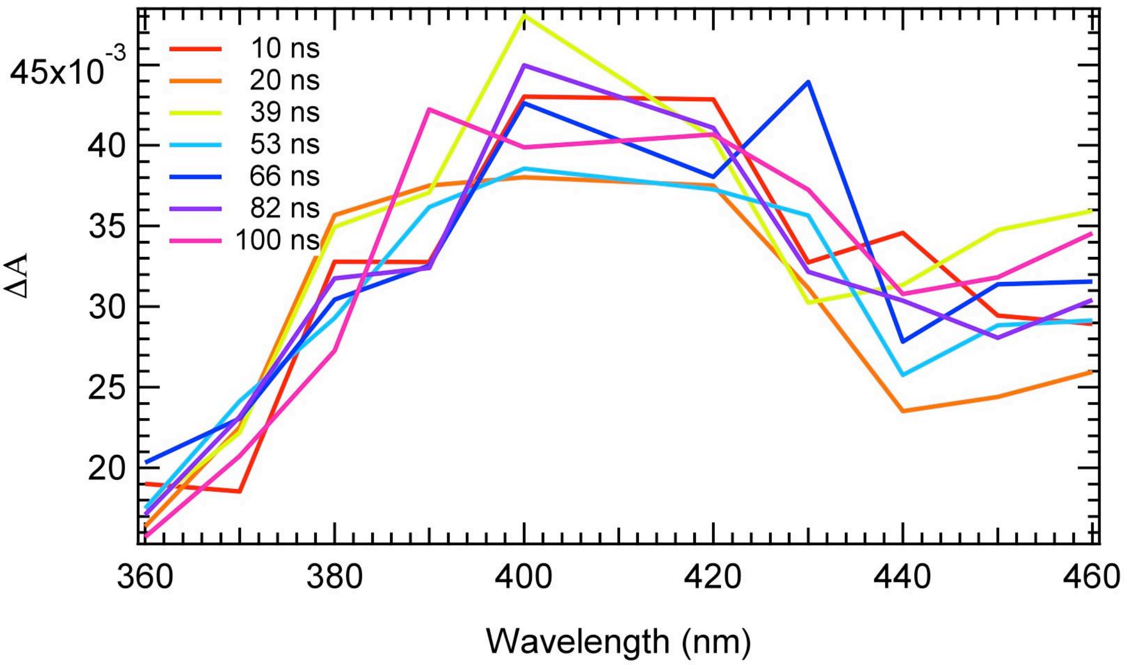

Supplementary Figure 2: There is no significant evolution in time-resolved difference absorption spectra recorded between 10 and 100 ns after a 410 nm nanosecond excitation of the *trans* protonated off-state of rsEGFP2 in H_2O solution (50 mM HEPES pH 8, 50 mM NaCl). The spectrum without laser excitation was subtracted to calculate the difference spectra.

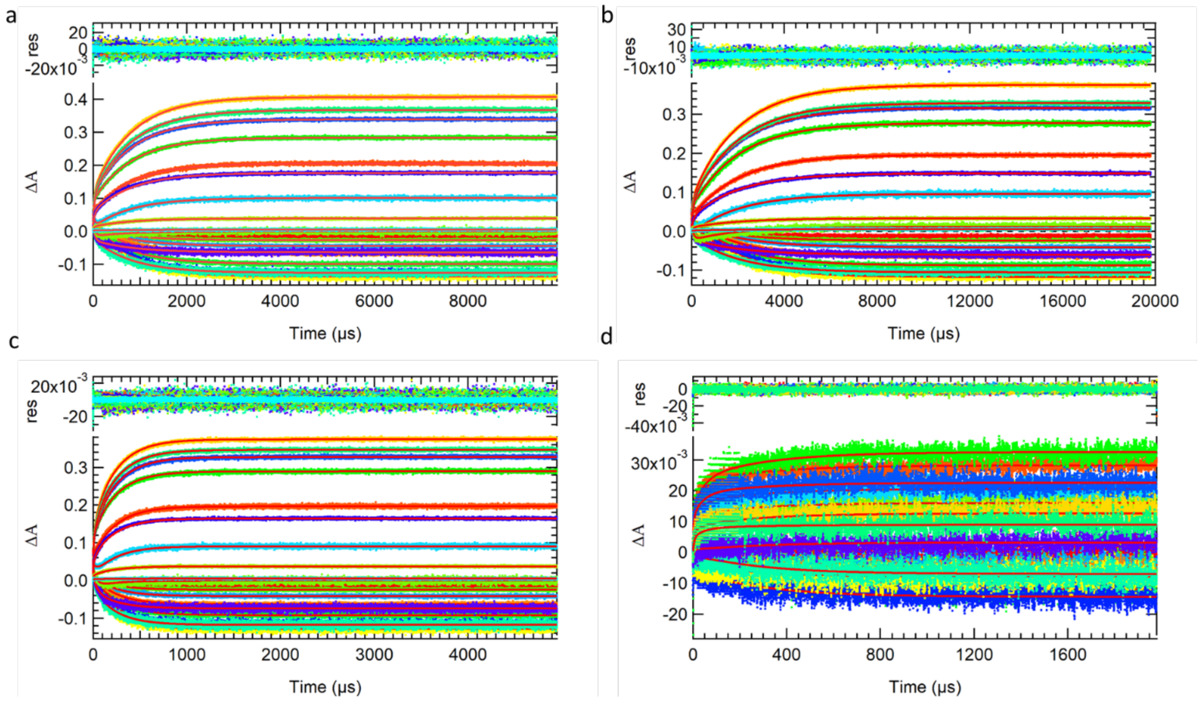

Supplementary Figure 3: Raw data (points in lower part of the panels) and global fit analysis (lines in lower part of the panels) with residuals (res; upper part of the panels) of the kinetic traces for 19 wavelengths (different colors, between 350 and 520 nm, 10 nm steps) with a weighted sum of three exponential decays for three rsEGFP2 solutions. (a) 50 mM HEPES pH 8, 50 mM NaCl in H_2O, (b) 50 mM HEPES pD 8, 50 mM NaCl in D_2O, (c) 50 mM HEPES pH 8, 50 mM NaCl, 1.25 M $(NH_4)_2SO_4$ in H2O) and (d) rsEGFP2 microcrystals in 100 mM HEPES pH 8, 2.5 M ammonium sulphate.

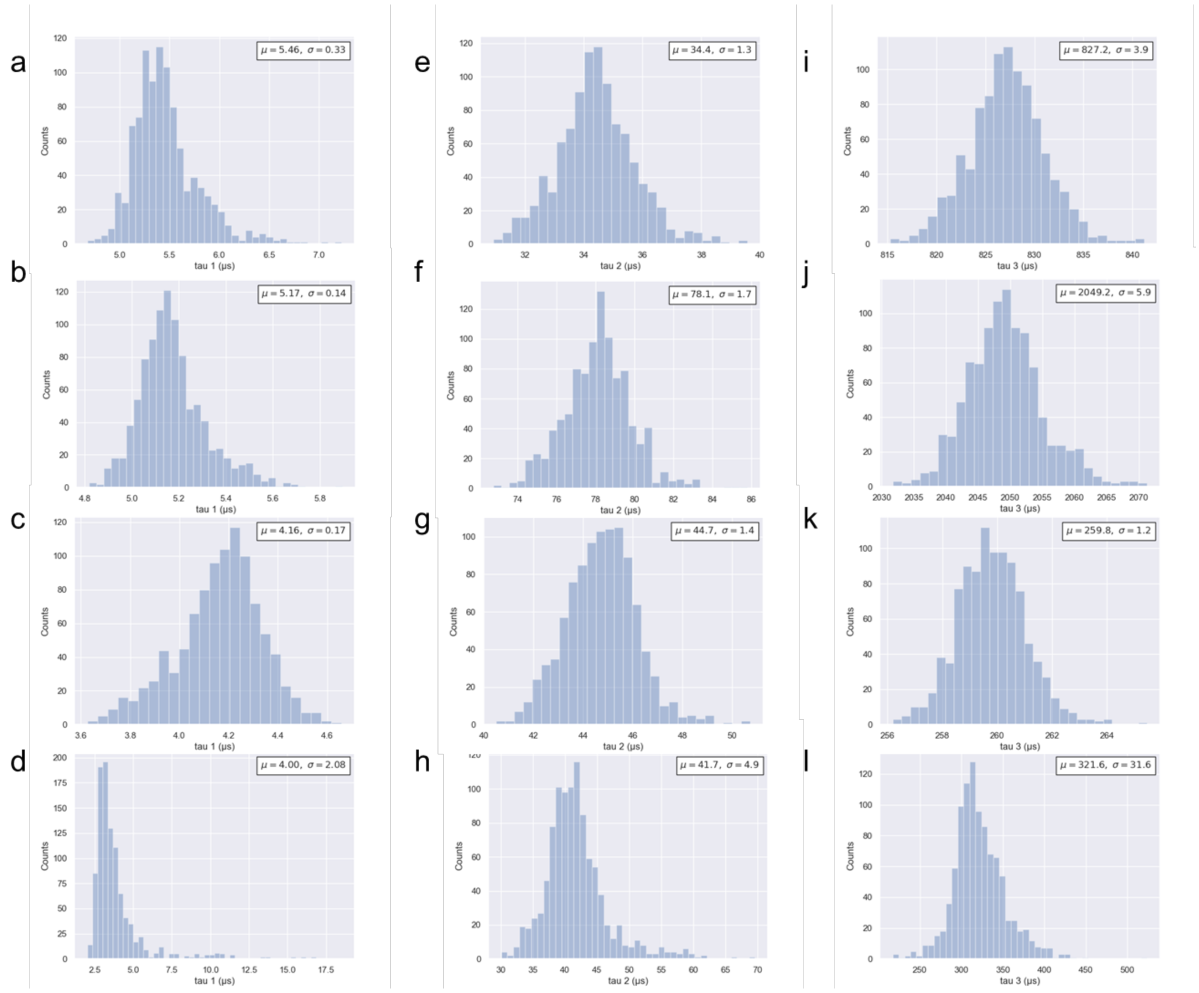

Supplementary Figure 4: Distribution of the three time constants retrieved for the 1000 replicates obtained by the bootstrapping procedure for rsEGFP2 solutions: (a, e, i) 50 mM HEPES pH 8, 50 mM NaCl in H_2O; (b, f, j) 50 mM HEPES pD 8, 50 mM NaCl in D_2O; (c, g, k) 50 mM HEPES pH 8, 50 mM NaCl, 1.25 M $(NH_4)_2SO_4$ in H_2O; (d, h, l) rsEGFP2 microcrystals in 100 mM HEPES pH 8, 2.5 M ammonium sulphate. Estimates of average μ and standard deviation σ are provided in microseconds.

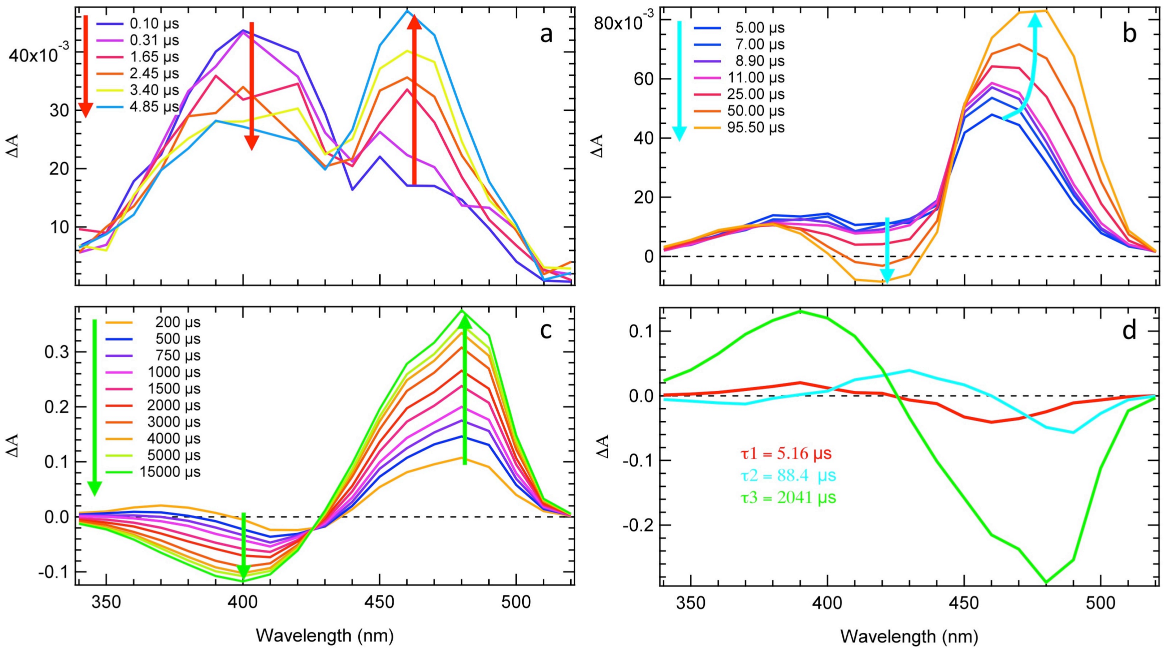

Supplementary Figure 5: Transient absorption spectroscopy of rsEGFP2 in D_2O solution (50 mM HEPES pD 8, 50 mM NaCl). Time-resolved difference absorption spectra recorded after a 410 nm nanosecond excitation of the *trans* protonated off-state in the time windows from 100 ns to 15 ms (*a* - *c*). The spectrum without laser excitation was subtracted to calculate the difference spectra. The coloured arrows (red in (a), cyan in (b) and light green in (c)) correspond to the three time constants (5.16, 88.4 and 2041 μs, respectively) identified by a global fit analysis of kinetic traces for all wavelengths. (*d*) Decay associated spectra obtained by fitting the kinetic traces in panels *a* – *c* for all wavelengths with a weighted sum of three exponential functions.

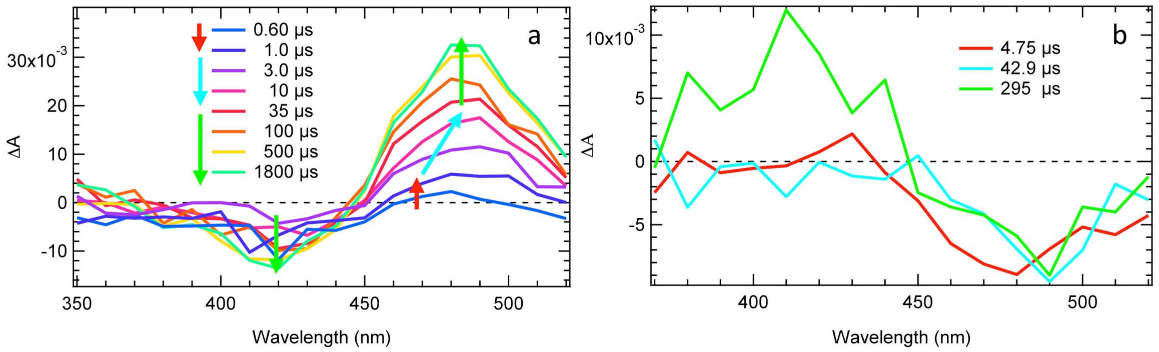

Supplementary Figure 6: Transient absorption spectroscopy of a suspension of rsEGFP2 microcrystals in 100 mM HEPES pH 8, 2.5 M ammonium sulphate. (*a*) Time-resolved difference absorption spectra recorded after a 410 nm nanosecond excitation of the *trans* protonated off-state. The spectrum without laser excitation was subtracted to calculate the difference spectra. The coloured arrows correspond to the three time constants identified by a global fit analysis of kinetic traces for all wavelengths. (*b*) Decay associated spectra obtained by fitting the kinetic traces in panel *a* for all wavelengths with a weighted sum of three exponential functions.

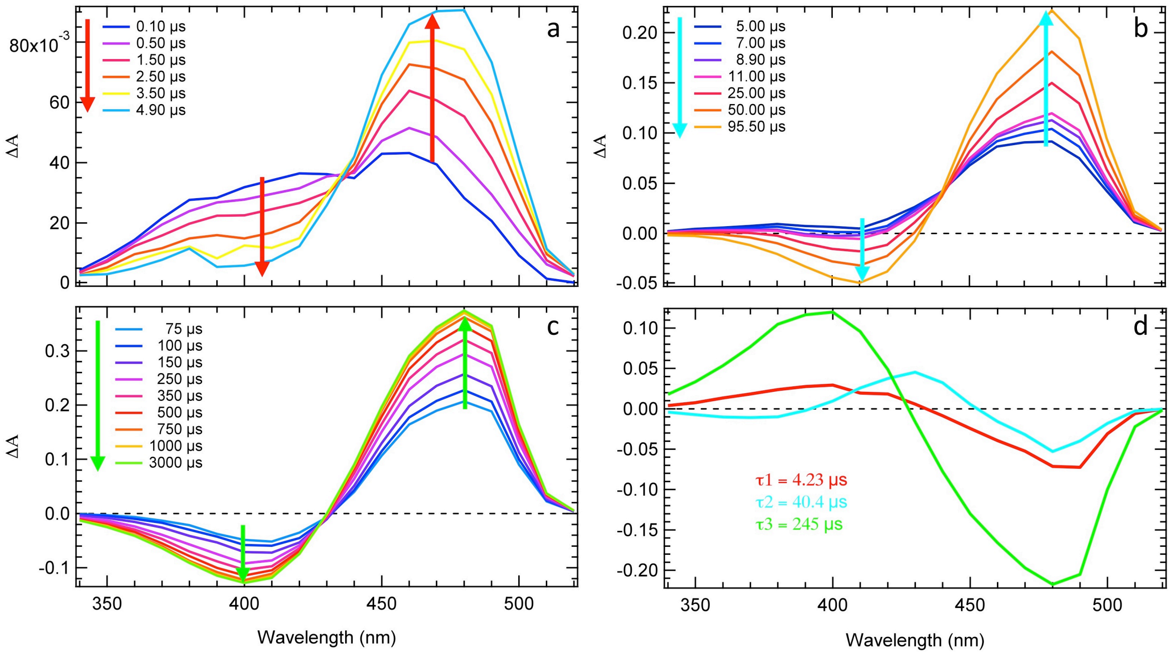

Supplementary Figure 7: Transient absorption spectroscopy of rsEGFP2 in H_2O solution (50 mM HEPES pH 8, 50 mM NaCl, 1.25 M ammonium sulfate). Time-resolved difference absorption spectra recorded after a 410 nm excitation of the *trans* protonated off-state in the time window from 100 ns to 3 ms (*a* - *c*). The spectrum without laser excitation was subtracted to calculate the difference spectra. The coloured arrows (red in (a), cyan in (b) and light green in (c)) correspond to the three time constants (4.23, 40.4 and 245 μs, respectively) identified by a global fit analysis of kinetic traces for all wavelengths. (*d*) Decay associated spectra obtained by fitting the kinetic traces in panels *a* – *c* for all wavelengths with a weighted sum of three exponential functions.

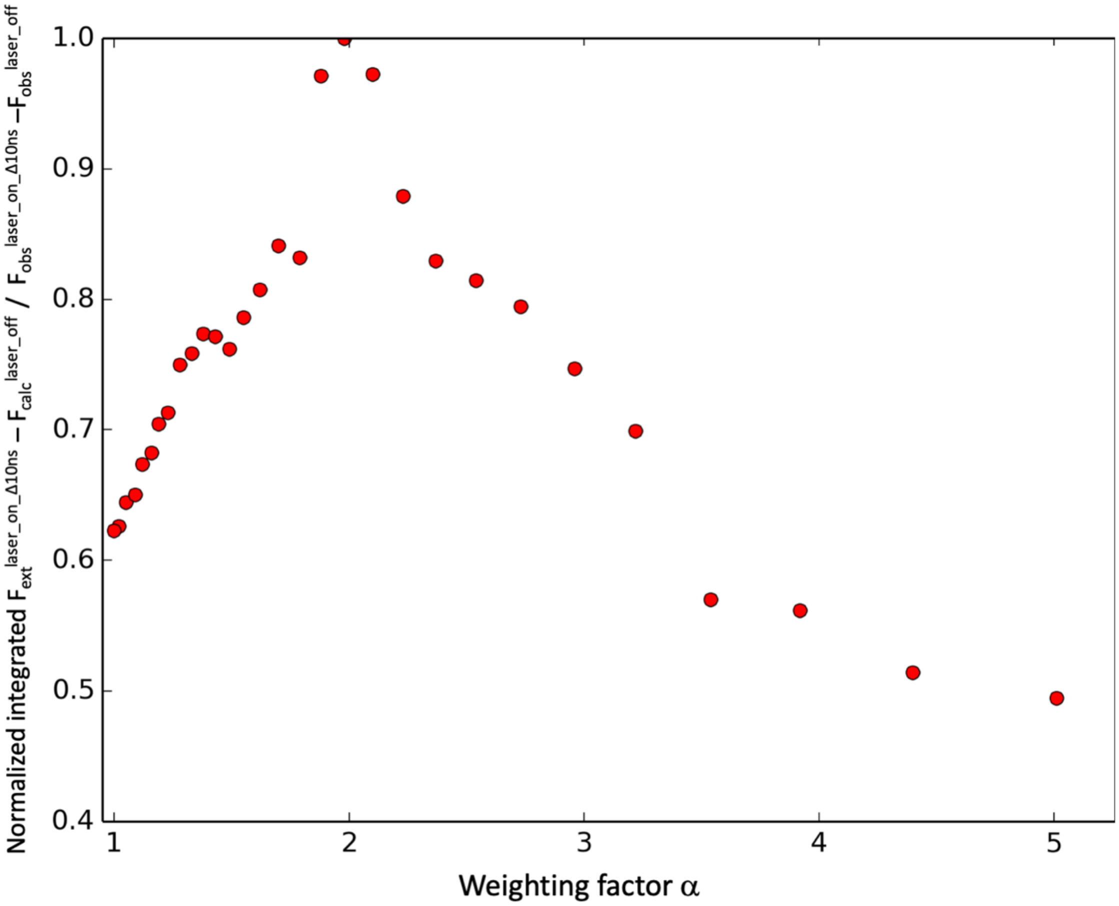

Supplementary Figure 8: Determination of the weighting factor α, corresponding to the inverse of the occupancy of the laser-on-Δ10ns model. The ratio of integrated peaks in the $F_{extrapolated}^{laser\_on\_\Delta 10ns} - F_{calc}^{laser\_off}$ map and of integrated peaks in the qW $F_{obs}^{laser\_on\_\Delta 10ns} - F_{obs}^{laser\_off}$ map, normalized to its maximum value, is plotted as a function of α. Only peaks around selected residues (chromophore, Tyr146, His149, Val151, and Thr204) were used for α determination.

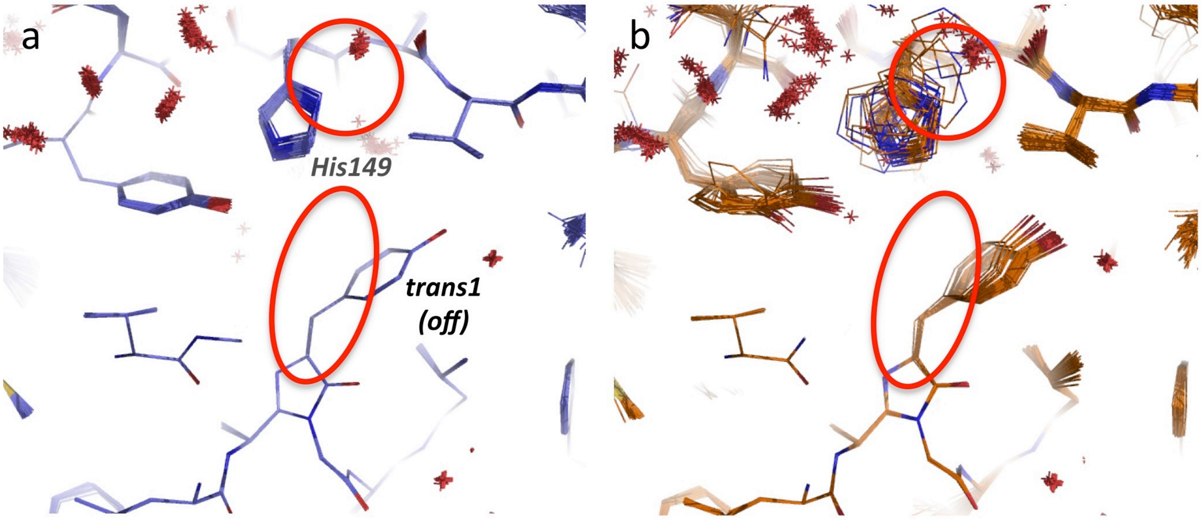

Supplementary Figure 9: Result of ensemble refinements against (*a*) the off-state data collected by cryo-crystallography at a synchrotron[18] starting from the corresponding off-state model (chromophore 100% *trans1*, PDB code 5DTY[18]) and against (*b*) the off-state data collected by room temperature SFX [27] starting for the corresponding off-state model (PDB code 5O8A) from which the *cis* alternate conformation was omitted so that the chromophore was fully in the *trans1* conformation [27]. No evidence is found for the occupancy of the *trans2* (oval contours) chromophore and His149-*supp* (circles) conformations.

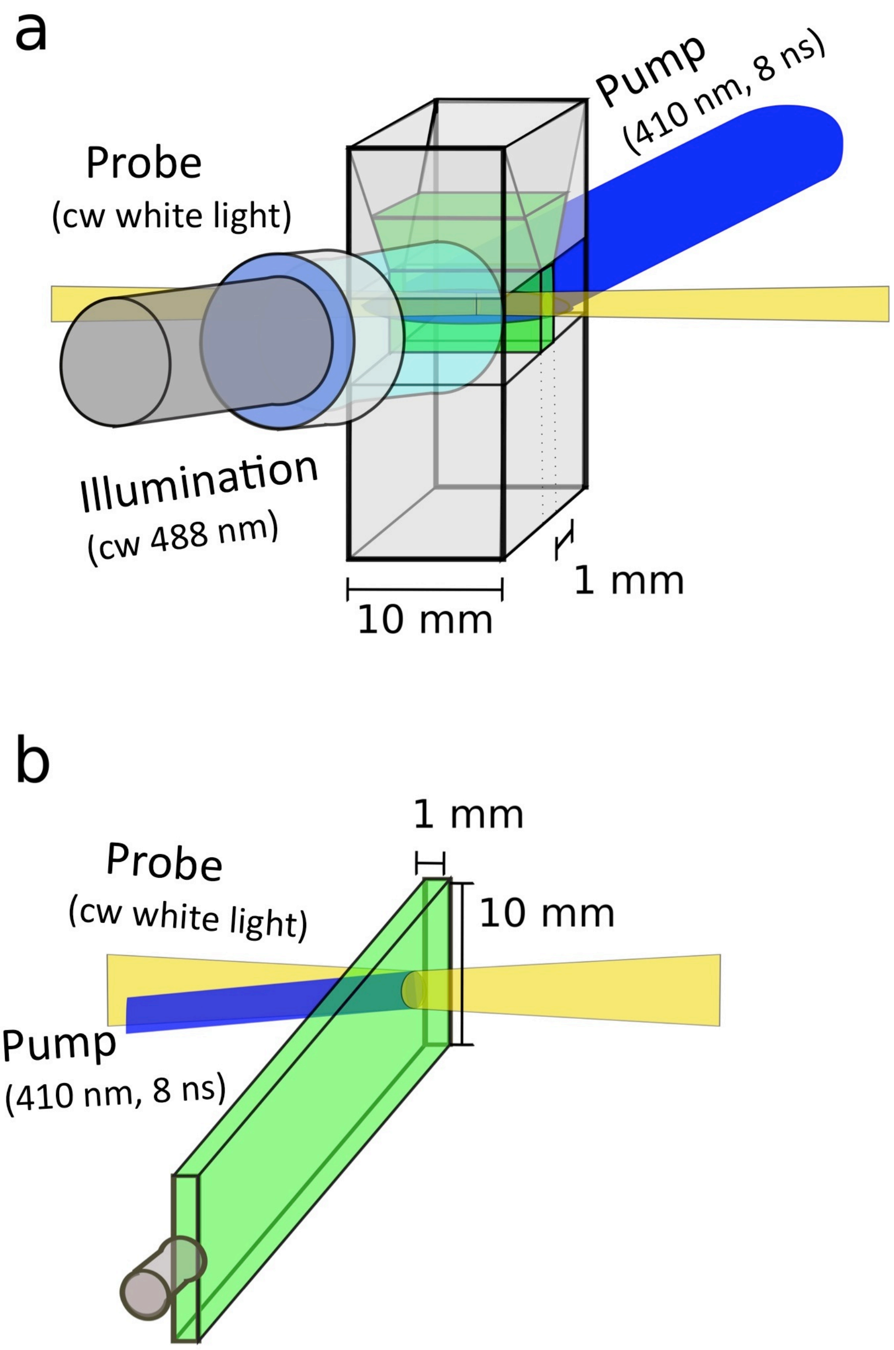

Supplementary Figure 10: Geometry between pump, probe and illumination beams at the spectroscopic cell for nanosecond time resolved absorption experiments of (*a*) protein solutions and (*b*) microcrystal suspensions.
